# Stem Cells From the Apical Papilla (SCAP) as a Tool for Endogenous Tissue Regeneration

**DOI:** 10.3389/fbioe.2018.00103

**Published:** 2018-07-24

**Authors:** Ola A. Nada, Rania M. El Backly

**Affiliations:** ^1^Oral Biology Department, Faculty of Dentistry, Alexandria University, Alexandria, Egypt; ^2^Tissue Engineering Laboratories, Faculty of Dentistry, Alexandria University, Alexandria, Egypt; ^3^Endodontics, Conservative Dentistry Department, Faculty of Dentistry, Alexandria University, Alexandria, Egypt

**Keywords:** stem cells from the apical papilla, angiogenesis, CD146, STRO-1, osteogenic differentiation, adipogenic differentiation potential

## Abstract

Stem cells extracted from developing tissues possibly exhibit not only unique but also superior traits against their developed counterparts. Indeed, stem cells from the apical papilla (SCAP); a unique group of dental stem cells related to developing roots have been shown to be a promising tool for regenerative endodontic procedures and regeneration in general. Studies have characterized the phenotypic traits as well as other regenerative potentials of these cells. Specific sub-populations have been highlighted as well as their neurogenic and angiogenic properties. Nevertheless, in light of the previously discussed features and potential applications of SCAP, there is still much to understand and a lot of information to unravel. The current review will discuss the role of specific markers for detection of different functional populations of SCAP; including CD146 and STRO-1, as well as their true multilineage differentiation potential. In particular, the role of the secretome in association with paracrine signaling in inflammatory microenvironments is also tackled. Additionally, the role of SCAP both *in vitro* and *in vivo* during regenerative approaches and in response to different growth factors and biologic scaffolds is highlighted. Finally, this review will shed light on current knowledge regarding the clinical translational potential of SCAP and elucidate possible areas for future research applications.

## Introduction—SCAP: description, histology, and fate

**Figure 1:**
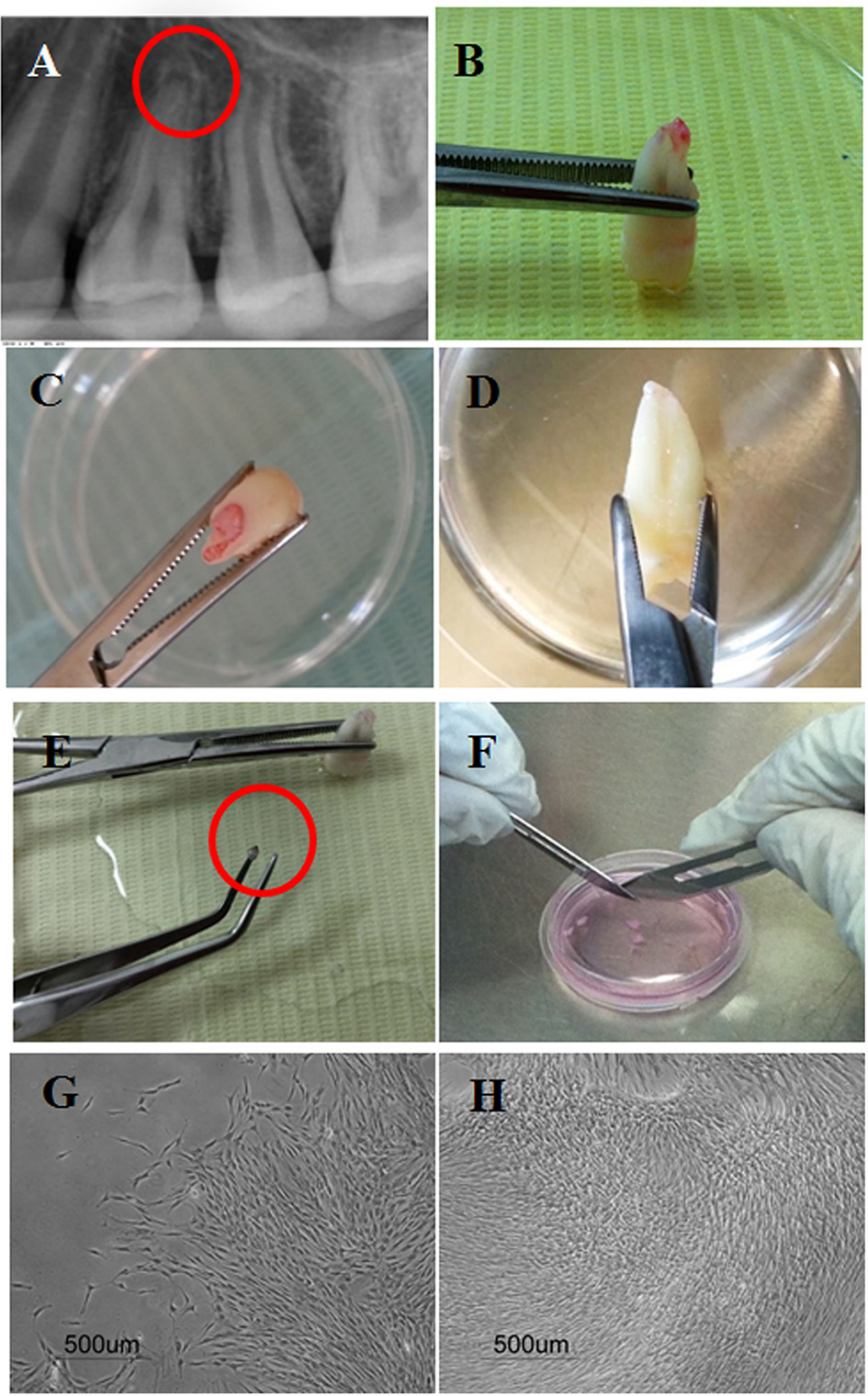
Isolation of stem cells from the apical papilla showing: **(A)** Periapical radiographs of the apical papillae related to the apex of a human premolar; **(B–D)** Extracted premolars with intact apical papillae. **(E)** Apical papilla separated gently using tweezers; **(F)** Scalpel dissection of apical papilla tissue in a cell culture dish with culture medium. **(G,H)** Cultured human SCAP at 4x after primary culture at 8 and 21 days, respectively.

Discovery of stem cells from the apical papilla (SCAP) was intrigued by the positive expression of a surface marker known as STRO-1, where STRO refers to mesenchyme; and thereby it is a marker used to detect mesenchymal stem cells (MSCs). The positive STRO-1 expression of SCAP was therefore taken as a clue that stem cells might be present in the apical tissues of teeth (Huang et al., 2008). As the name implies, these stem cells are found at the apical papilla of the tooth. This papilla tissue is basically the apical part of the formerly known dental papilla which was the mesenchymal counterpart involved in the epithelial mesenchymal interactive process that led to tooth development (Nanci, 2012). Being the apical part of the dental papilla makes it present after at least two thirds of the root has formed. Histologically, the apical papilla can be seen precisely apical to the epithelial diaphragm, with a cell rich zone separating it from the dental pulp. Despite both the dental pulp and apical papilla being basically a continuity of one another, when necrosis strikes the pulp, the apical papilla combats the pulp and tends to survive due to its accessibility to a collateral circulation apically (Huang et al., 2008; Diogenes and Hargreaves, 2017). Therefore, justifying how immature teeth with necrotic pulps are able to undergo completion of root development/ apexogenesis sometimes even with persistence of signs and symptoms indicative of apical periodontitis; further elucidates the infection-resistant nature of SCAP (Chrepa et al., 2017; Lin et al., 2018). The apical papilla and consequently SCAP can be easily isolated following tooth extraction by separating the tissue at the tips of the developing roots by tweezers. The tissue is then dissected into smaller pieces, and digested using a cocktail of collagenase and dispase in a well-established protocol to isolate single cell suspensions which are then cultured (Huang et al., 2008) (Figure 1).

**Figure 2:**
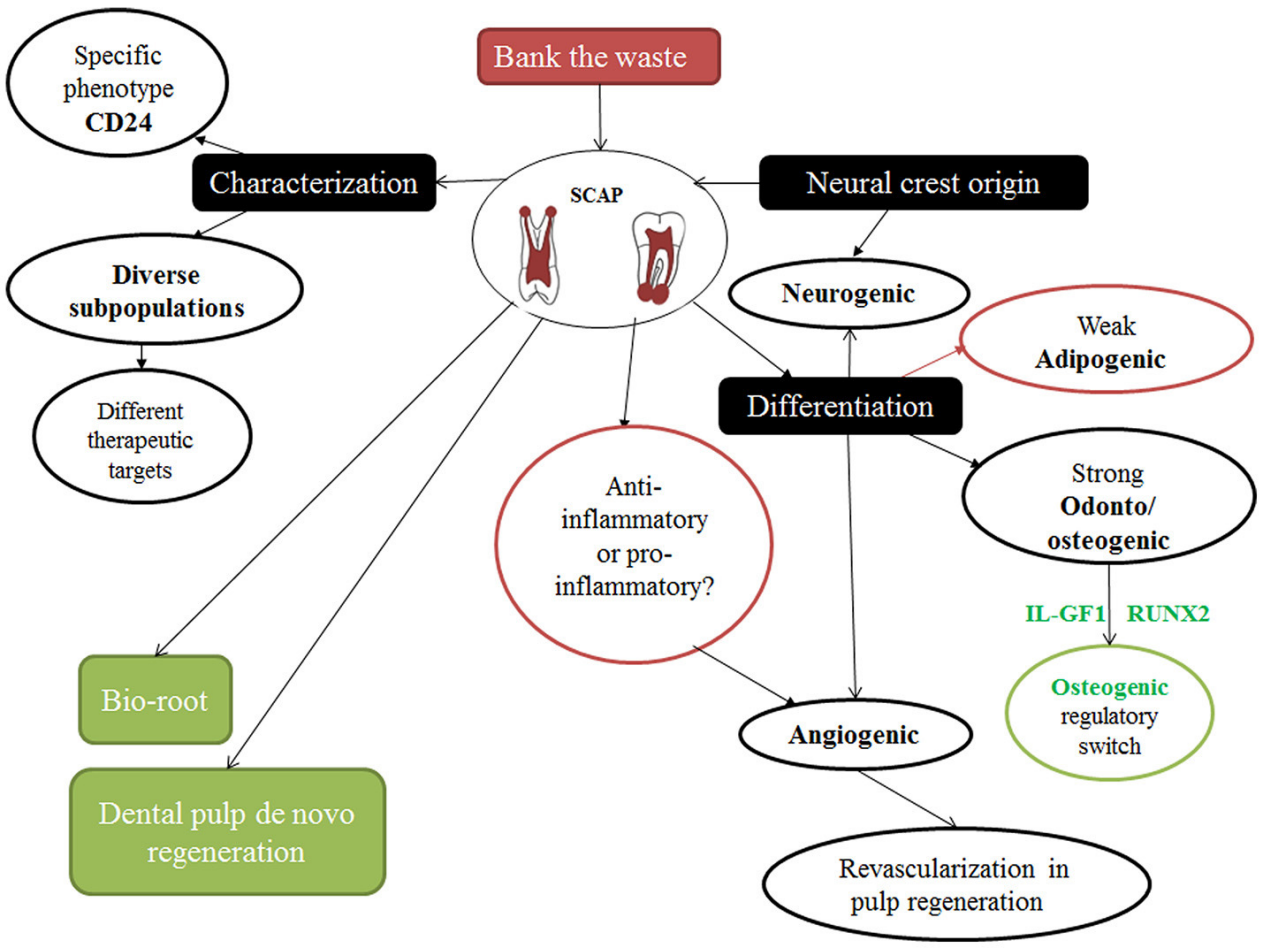
Cartoon showing different particular features of SCAP and their implications.

It is worth mentioning that without the presence of SCAP, root maturation ceases to continue (Huang et al., 2008; Sonoyama et al., 2008; Zhang et al., 2014, 2015). Furthermore, SCAP have been characterized by their plasticity, potency, and versatility as a dental stem cell (DSC) group in superior contrast to the infamous dental pulp stem cells (DPSCs) (Yuan et al., 2014) (Figure 2).

## Phenotypic characterization of SCAP and their therapeutic implications

Flow cytometric characterization is routinely used to detect the expression of various stem cell surface markers. Different SCAP phenotypes have been suggested to correlate to distinct functional properties (Bakopoulou et al., 2012). Research has shown that MSCs can be found in vascular tissues (Lv et al., 2014). Reflecting the perivascular location of SCAP is their positive expression of stem cell (SC) markers such as STRO-1 and CD146, with both expressions fading with passaging (Bakopoulou et al., 2012; Schneider et al., 2014; Liu et al., 2016). Moreover, SCAP also positively express CD73, CD90, and CD105; typical MSC markers (Hilkens et al., 2017).

Of the various cell surface markers, CD24, a marker of pluripotency has been found to be directly correlated to SCAP; where it was found to be exclusively expressed in SCAP (Liu et al., 2016). Even though it is expressed at low amounts ranging from 3.2 to 15% (Ding et al., 2010; Chen et al., 2012; Dong et al., 2013), it is considered to be SCAP specific since it has been found to be undetectable in other MSCs, including DPSCs. CD24 is actually used as an indicator of undifferentiation, and hence its presence indicates an increased stemness of cells. This was proven by the finding that as alkaline phosphatase (ALP) level increases, CD24 expression level decreases, denoting that the cells have started leaving the state of undifferentiation and entering that of the osteoblastic lineage (Chen et al., 2012; Dong et al., 2013).

On the other hand, another study showed that regardless of differentiation, once cells reach the 10th passage, their expression of CD24 decreases to 0%. Seeing as though CD24 is considered an identifying marker of SCAP, this finding is rather interesting as it possibly implies that the stemness of SCAP fades with passaging (Zhang et al., 2014).

Furthermore, on the topic of positive expression of surface markers is the positive expression of the perivascular multipotent stem cell marker CD146/ MUC18 that is normally expressed in human endothelial cells. Its positive expression on SCAP has been found to be quite controversial where the literature describes its expression to range between 47 and 85% (Sonoyama et al., 2006; Bakopoulou et al., 2013; Zhang et al., 2014). A unique finding was SCAP showing greater expression peaks of CD146 above the range limit, where expression was sometimes found at levels greater than 90% (Bakopoulou et al., 2015; Table 1).

**Table 1:** Demonstrates the percentage of CD146 and STRO1 positive SCAP cells obtained from human premolars.

**Samples**	**Percentage of marker expression (%)**			
**Number**	**CD146 (lymphocyte gate)**	**STRO1**	**CD146, STRO-1 (co-expression)**	**CD146 (large -sized cells; granulocyte gate)**
1	7	10	6	49
2	5	13	11	48
3	1	6	0.87	23
4	4	11	9	20
5	20	1	0.5	64
6	7.4	0.15	5.5	92.7

*The percentage of marker expression for CD146 is displayed in both lymphocyte and granulocyte gates showing different CD146+ cells based on their physical characteristics and marker expression*.

Regarding SCAP populations co-expressing the perivascular markers CD146 and STRO-1, we have found in our work much lower percentages as compared to those documented in the literature (Sonoyama et al., 2006; Bakopoulou et al., 2013; Yuan et al., 2014; Zhang et al., 2014; 0.5–11%; Table 1). This could partly be owing to the source of cells being maxillary premolars extracted for orthodontic purposes as compared to the most commonly documented source in the literature; wisdom teeth (Sonoyama et al., 2006, 2008; Huang et al., 2008; Bakopoulou et al., 2012, 2013; Wang et al., 2013; Yuan et al., 2014; Zhang et al., 2014, 2015). This finding quite possibly negated our hypothesis that SCAP obtained from teeth at a younger age range suggests a greater MSC expression. However this conclusion cannot be made due to the various confounding variables available such as cell passage, epigenetic changes that occur with culturing, microenvironment changes, exact time of trypsinization for flow cytometry processing, and the technical procedure of flow cytometry itself (Bakopoulou et al., 2011; Lv et al., 2014).

The significance of the expression of CD146 lies in it being the most utilized chief marker to characterize perivascular multipotent stem cells in connective tissues. However, like all markers, CD146 expression is not homogenous, and therefore requires fine tuning/ sorting in order to get a pure population. Moreover, due to its variable initial expression level, like STRO-1, neither STRO-1 nor CD146 can be utilized alone as a dental MSC specific markers (Bakopoulou et al., 2013; Lv et al., 2014).

**Figure 3:**
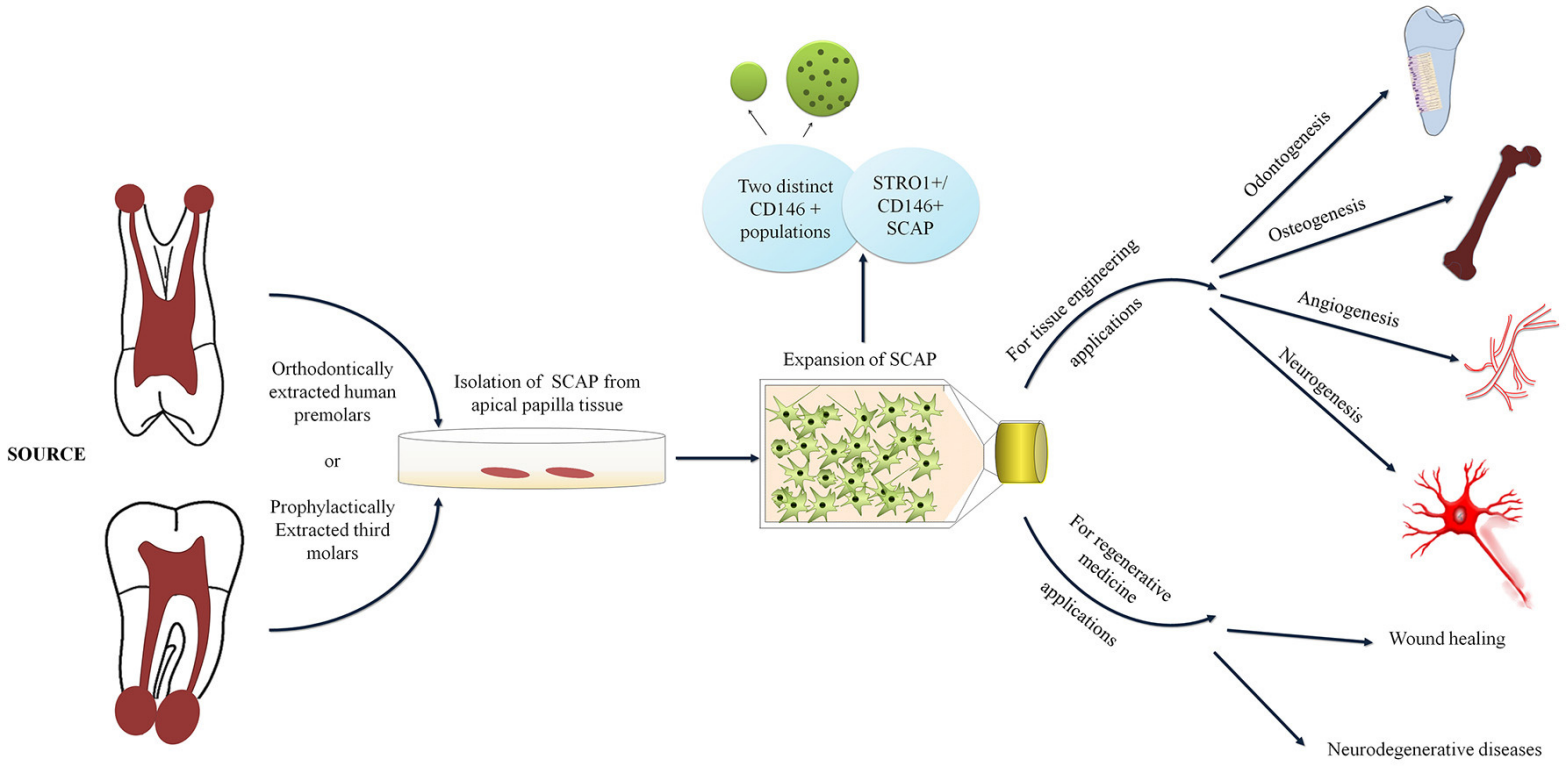
Diagram depicting SCAP cell surface characteristics and applications in tissue engineering and regenerative medicine.

Contrary to the majority of research undertaken, we have seen that our isolated SCAP populations have lower percentages of CD146 in contrast to STRO-1 (Table 1; Sonoyama et al., 2006; Bakopoulou et al., 2013; Yuan et al., 2014; Zhang et al., 2014). Nonetheless and in compliance with Bakopoulou et al, two separate populations expressing CD146 were detected (De Berdt et al., 2015). However, Bakopoulou et al. did not elaborate on the characteristics of the 2 distinct CD146 populations other than mentioning that the expression of CD146 in the other population was remarkably high. This was in accordance with what we have also seen in premolar cultures, where the additional CD146 positive population yielded upper limit expressions. We have found that these cells are not only larger but also more granular as this distinct population appears to be identified in the granulocyte gate upon flow cytometric analysis (Figure 3). Moreover, as hypothesized by other studies, it is also possible that these cells represent perivascular precursors rather than MSC populations (de Souza et al., 2016). Thereby, providing an interesting possibility of rendering SCAP as a population that houses cells having a rather rich perivascular origin and therefore opening up wider angiogenic options.

Bakopoulou et al. did however report that when SCAP were sorted to being positive for STRO-1 and CD146 only, cells were observed in fact morphologically bigger with greater granularity (Bakopoulou et al., 2013). However, even though the additional population with CD146 positive cells in our experience were also larger and more granular, as identified in the granulocyte gate upon flow cytometric analysis, they strikingly did not exhibit higher positive co-expression with STRO-1. On the contrary, the large sized CD146 positive population had very low expression of STRO-1 highlighting the possible presence of a distinct and separate cell population (Table 1).

Further contradicting with Bakopoulou et al. were when SCAP was sorted into being only positive for CD146 and negative for STRO-1, cells were seen to be smaller with lesser granularity despite the positivity of CD146 (Ding et al., 2010). This might once more possibly suggest a possibility to unravel other existing subpopulations with special features, predictably involving angiogenesis and endothelial cell activity seeing as though CD146 mainly characterizes perivascular multipotent stem cells (Gosau et al., 2013).

Another sub/side population in SCAP has been found to predominantly express STRO-1. These STRO-1 positive cells were found to encompass neurogenic characteristics, seeing as they stained positively for beta III Tubulin, glutamic acid decarboxylase (GAD), nestin, and neuron specific enolase; all of which are neuronal stem markers (Huang et al., 2008). This confirms the notion, that according to the percentage of positivity of distinct MSC markers, cells become more oriented toward certain lineages. Furthermore, light has been shed upon the significance and correlation of STRO-1 expression generally and stem cell properties, to the extent that raw heterogeneous, i.e., non-sorted populations were found to be superior to sorted ones due to the presence of STRO-1 (Bakopoulou et al., 2013).

Furthermore, a sorted SCAP population with double expression of STRO-1 and CD146 has been implicated to be of a superior quality. This implication came forward when double positive cells showed not only better stem cell properties such as colony forming efficiency (CFE), odontogenic differentiation potential, and mineralization capacity, but also expressed various critical embryonic markers such as Nanog, Oct 3/4, SSEA 3, TRA1-60, and MSC markers. Of the increased expression of the pluripotency markers, TRA-1-60, an early embryonic marker was not detectable at all in the unsorted population to begin with. It was brought to detectable levels after sorting the population into being double positive for CD146 and STRO-1 (Bakopoulou et al., 2013).

Despite the successful coexistence of STRO-1 and CD146, their co-expression is not considered a requirement for the characterization of SCAP. Nevertheless, it has been initially concluded that a smaller but uniform subpopulation is more likely to function superiorly than a larger more diverse population. (Bakopoulou et al., 2013). In spite of this, more research is required to tackle all variables at hand and ensure that it is primarily the role of the population sorting that produces certain effects and no other variables as well.

Finally, SCAP have been found to simultaneously minimally or negatively express CD14, CD31, CD34, CD45, and HLA DR (Huang et al., 2009; Cao et al., 2013; Schneider et al., 2014; Hilkens et al., 2017). The negative expression of the leukocyte precursor CD45 is particularly vital as it confirms the stromal origin of SCAP and the absence of hematopoietic precursor contamination (Sonoyama et al., 2006; Huang et al., 2009; Bakopoulou et al., 2011, 2012; Li et al., 2014). Therefore, for efficient stem cell (SC) characterization, besides the basic International Society for Cellular Therapy (ISCT) criteria of positively expressing a group of antigens and negatively express others. The former being CD105, CD73, and CD90, and the latter being CD45, CD34, CD14 or CD11b, CD79a or CD19, and HLA-DR. (Lv et al., 2014). Furthermore, SCAP can specifically be characterized by the additional expression of CD24 and SCAP properties can be enhanced/altered by sorting the cells in accordance to STRO-1 and CD146. For instance, for studies targeting bone regeneration potential, it is recommended to preselect populations that are double positive for CD146 and STRO-1 (Bakopoulou et al., 2013), whereas when neurogenic regeneration is targeted, STRO-1 positive populations should be preselected (Huang et al., 2008). In another instance when pluripotency is targeted, CD24 positive populations should be selected (Liu et al., 2016).

## Classical tri-lineage mesenchymal potential—role of SCAP in developmental biology reflected *in vitro*: potent osteogenesis and weak adipogenesis

SCAP have been proven to differentiate when stimulated by respective inductive media to osteo/odontoblast like cells, adipocytes, as well as chondroblasts (Zhang et al., 2014).

### Strong osteogenic differentiation potential

Osteogenic/odontogenic differentiation potential of SCAP has been shown to be successful through positive alizarin red S staining of formed calcific deposits, in addition to the corresponding upregulation of mineralization markers as Dentin sialophosphoprotein (DSPP), Bone sialoprotein (BSP), and ALP. To a lesser extent, an increase in Bone gamma-carboxyglutamate protein (BGLAP), Bone morphogenetic protein (BMP), and Runt-related transcription factor 2 (RUNX2) has also been noted (Bakopoulou et al., 2013).

Compared to Bone Marrow Mesenchymal Stem Cell (BM MSCs), SCAP differentiation along the osteogenic line has been found to be quite comparable (Dong et al., 2013). Not only comparable, but both SCAP and DPSC seem to be superior to BM MSC in their osteo/odontogenic differentiation potential (Sonoyama et al., 2008; Huang et al., 2009).

It is worth mentioning that the expression of the osteo/odontogenic markers Dentin Sialoprotein (DSP), ALP, BSP, and Osteocalcin (OCN) was found in odontoblasts lined against newly formed dentin yet were not expressed in the apical papilla source itself (Sonoyama et al., 2008). This highlights and further emphasizes the favorable undifferentiated state of the apical papilla source.

**Figure 4:**
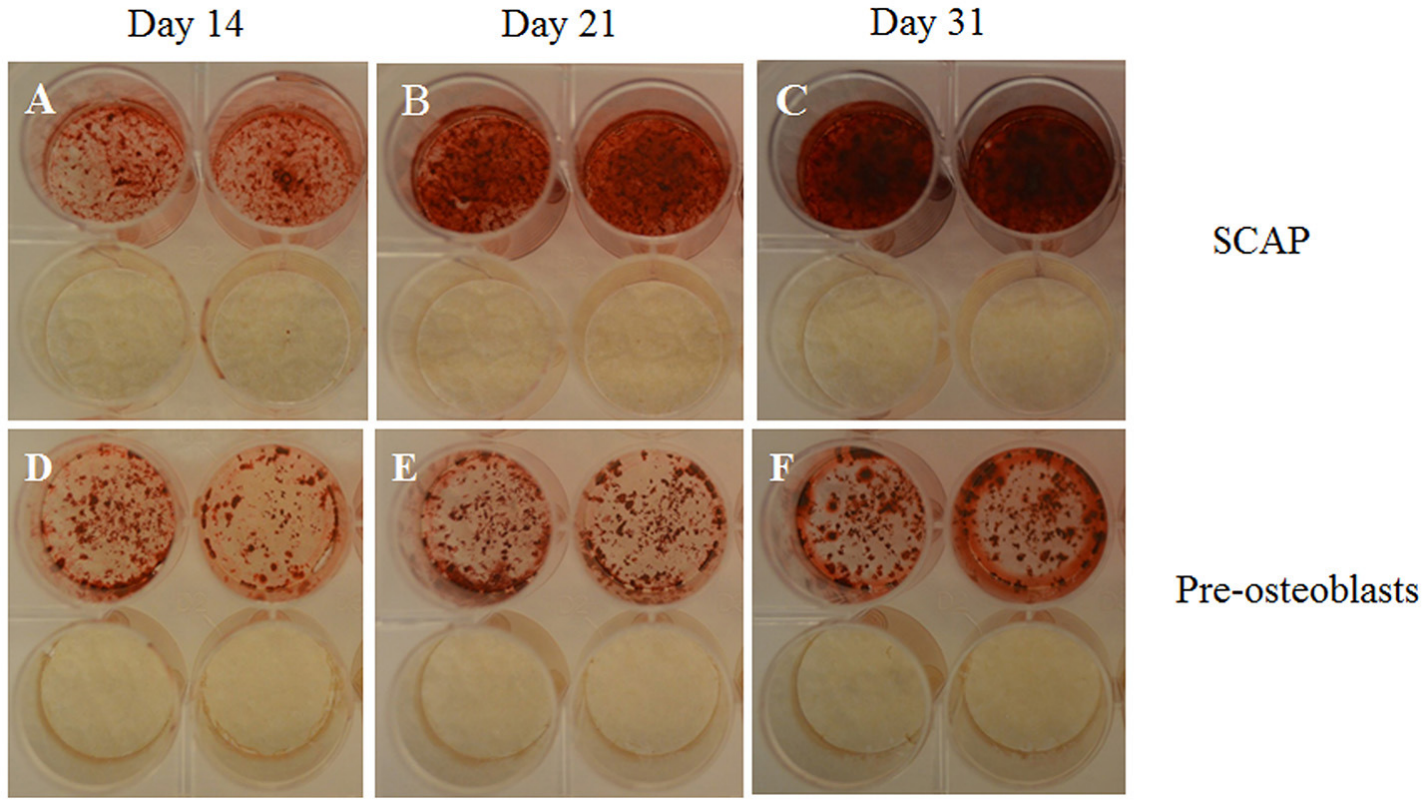
Photographs of wells; taken from 24 well plates showing the more intense Alizarin Red S stain uptake by SCAP **(A–C)** vs. preosteoblasts **(D–F)** along 3 time intervals; 14 days **(A,D)**, 21 days **(B,E)**, and 31 days **(C,F)**. Note the lack of stain uptake in the non-induced wells in both cell types. Osteogenic differentiation protocols used were in accordance to Sonoyama et al. (2008).

**Figure 5:**
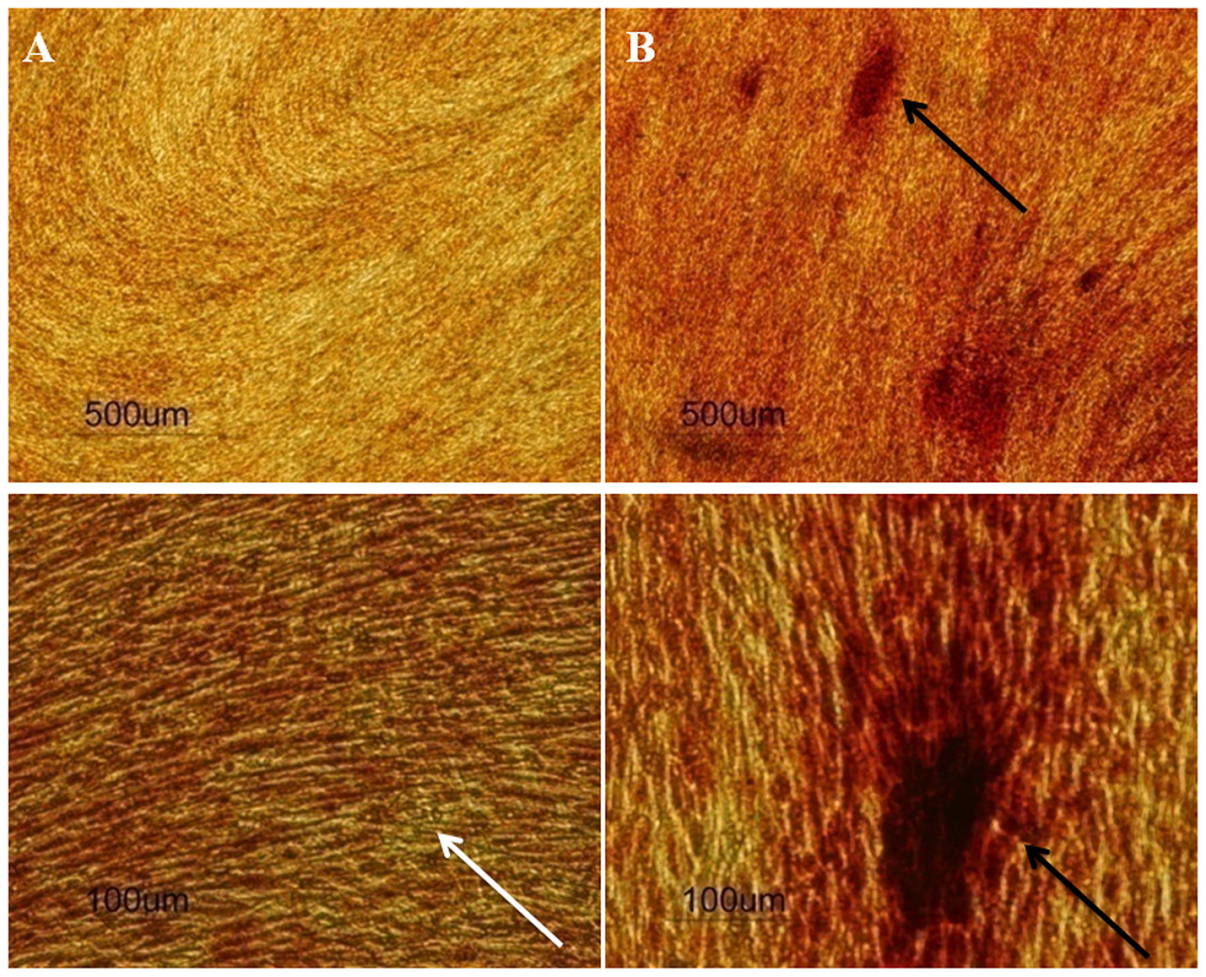
Human premolar SCAP cultures stained by Alizarin Red S stain after **(A)** 14 days and **(B)** 21 days of osteogenic induction (*n* = 20). Upper row: 4x, lower row 20x magnification. The white arrow shows diffuse calcifications at day 14, while the black arrows show discrete intense calcifications at day 21.

Moreover, SCAP are rather committed to osteogenic differentiation, seeing as though upon stimulation, cells differentiated readily into osteoblast-like cells and produced Alizarin Red S stained calcific deposits (Figure 4). Different patterns of differentiation were also noticed according to the source of SCAP, where premolar cultures gave rise to more localized calcific deposits as passage progressed, whereas wisdom cultures gave more diffuse calcifications as passages progressed. The explanation for this difference is not clear and requires further confirmation and elucidation. Nevertheless, Volponi et al. using Raman spectroscopy described the composition of the mineralized material yielded by SCAP as discrete accumulation (Volponi et al., 2015) which is similar to the mineralization pattern resulting from our premolar cultures (Figure 5).

Furthermore, to confirm the specificity of the Alizarin red S staining, parallel positive and negative controls were conducted. Positive controls being mouse preosteoblasts (MC3T3-E1) and negative controls being malignant peripheral nerve sheath tumor cells (MPNST). Rather unexpectedly, human SCAP showed greater mineralization potential in contrast to the already committed mouse preosteoblast cell line, reinforcing SCAPs' mineralization potential upon osteogenic differentiation (Figure 4).

In favor of SCAP, both Sonoyama et al. and Huang et al, reported that SCAP osteogenesis is superior to BM MSC osteogenesis (Sonoyama et al., 2008; Huang et al., 2009; Schneider et al., 2014) Therefore, coupling their remark with our positive osteogenic results concludes that SCAP would serve as the optimal stem cell of choice when attempting experiments involving osteogenic differentiation or when tackling bone regeneration in general. This conclusion is highly supported due to the ease by which SCAP are isolated contrary to the invasive and painful isolation of BM MSCs. Although DPSCs and SCAP appear to have similar potentials, extraction of the apical papilla tissue from root tips is much simpler and more convenient than from the dental pulp tissue which involves careful sectioning of the tooth. Thereby, making SCAP a more feasible source of MSCs.

To elaborate, in terms of the osteogenic supplementation used, basic supplements are agreed upon and do guarantee calcific deposit formation. These supplements include, Dexamethasone, β-glycerophosphate and ascorbic acid. In our experiments, the previously described function of ascorbic acid was strongly confirmed, where control/ non-induced cultures with basal media containing ascorbic acid did in fact show uniform Alizarin Red S staining post differentiation. Thereby, confirming its aforementioned function of stimulating the secretion of Collagen type I (Cao et al., 2013).

Various studies showed that both proliferation and osteogenic/odontogenic differentiation capacities can be enhanced by certain media supplements, for instance adding KH2PO4 or replacing fetal bovine serum (FBS) with 5% human platelet lysate (PL), as well as providing BMP signaling (Gao et al., 2013; Wang et al., 2013; Na et al., 2016). The human alternative of FBS being PL especially poses great opportunities for *in vivo* trials, since platelet lysates represent more predictive markers of the possible *in vivo* microenvironment (Wang et al., 2013).

Furthermore, an overall superior alternative to using osteogenic supplements altogether, is the use of amniotic membrane (AM) to stimulate osteogenic differentiation of SCAP. This is particularly relevant when considering *in vivo* osteogenic induction, AM seems to have better chances of *in vivo* retention in contrast to the more soluble osteogenic supplements (Chen et al., 2012).

Besides the chemical combinations or materials the cells are exposed to, methodologically speaking, protocols varied among studies in the sense of the time frame of osteogenic induction and any preconditioning the cells received. For instance, in some studies, cells were subjected to overnight serum starvation post confluence prior to osteogenic induction (Na et al., 2016). This difference possibly places cells in a state of stress, thereby stimulating their innate expression of relevant growth factors. Other studies induced osteogenesis after 24 h rather than at confluence (Gao et al., 2013). These differences however do not seem to pose significant implications.

#### SCAP directions; a dental tissue with a regulatory switch between odontogenic and osteogenic routes

Most studies seem to combine between the two differentiation routes of osteogenic and odontogenic differentiation due to the overall similarities of the two tissues. These similarities are not only restricted to their composition, but also to their matrix mediated mineralization mechanism of formation, in which type I collagen generates the structural template for the epitaxial nucleation of hydroxyapatite (HA) (Wang et al., 2012). However, despite the known similarities between bone and dentin, marked differences are present, whether it be in the histology or the related molecular biology of each of the two tissues.

Few studies pointed out to variables such as Insulin-like growth factor 1 (IGF-1) and Runt-related transcription factor 2 (RUNX2) that could act as directors as to which route SCAP follows. For instance, treatment of SCAP with IGF-1 potentiated osteogenesis through elaboration of bone specific proteins; Osterix (OSX) and Osteocalcin (OCN).Whereas, when SCAP were left untreated with IGF-1, rather than the expected simple decrease in osteogenesis, an increase in the odontoblastic specific marker DSP was noted. Hence, a switch was observed, where instead of bone-like tissue generation, dentin-pulp like tissue was generated. This concluded that treating SCAP with IGF-1 leads to osteogenesis, whereas not treating SCAP with IGF-1, led to dentinogenesis (Wang et al., 2012).

What is worth mentioning is the expression of OCN, which was surprisingly upregulated in the odontoblast-like cells of the non-treated SCAP. This upregulation was more or less focused at the odontoblastic cell layer, where its expression was found to be less in the osteodentin-like tissue and then obsolete in the dentin-like structure (Wang et al., 2012). A possible explanation might be the initial similarity between the formative osteoblast-like and odontoblast-like cell vs. the differences in each of their secretory products. Moreover, a common and interesting finding for both treated and untreated SCAP was the expression of DSPP, which was found to be rather low in either case (De Berdt et al., 2015). This might be due to the fact that SCAP is from a developing tissue and might therefore not have the capacity to fully achieve odontoblastic differentiation *in vitro* due to the lack of the supportive *in vivo* microenvironment.

A more recent study has shed some light on the more recognized master transcription factor, RUNX2, suggesting that a diversion from osteoblastic to odontoblastic differentiation route was noted when the expression of RUNX2 was downregulated (Bakopoulou et al., 2013).

Micro environmental conditions such as the pre-existence of infection also seem to influence the mineralization capacity of SCAP. In a recent study, SCAP were subjected to different concentrations of lipopolysaccharides (LPS) extracted from Porphyromonas gingivalis (Lertchirakarn and Aguilar, 2017). Even though seemingly lower concentrations of LPS did not adversely affect neither proliferation nor differentiation potential of the cells, higher concentrations (5 μg/ml) actually resulted in a significant upregulation of BSP gene expression thereby causing a more pronounced osteogenic response. This may partially explain the fact that the most frequently encountered tissues within the canals of immature necrotic teeth treated with regenerative endodontic procedures appeared to be either bone-like or cementum-like in nature (Nosrat et al., 2015). It appears that while SCAP can indeed survive the presence of infection, their nature may however be influenced by the microenvironment thereby dictating the type of regenerated tissues (Yoo et al., 2016). Unfortunately, the presence of bacterial biofilms not effectively eradicated by chemical disinfection regimens may have detrimental effects on the dentinogenic and osteogenic capacities of SCAP warranting a continued search for appropriate irrigants and intracanal medicaments that are effective while at the same time respecting stem cell biology (Vishwanat et al., 2017).

### Weak adipogenic differentiation potential

Several studies have proven SCAPs' adipogenic potentiality through the confirmatory oil red O staining of formed oil droplets following adipogenic induction. More thoroughly, gene expression of Lipoprotein lipase (LPL) was also noted to be at a high level confirming adipogenesis and the presence of lipid rich vacuoles (Bakopoulou et al., 2013).

Compared to BM MSCs, SCAP adipogenesis was found to be of a lower quality since only a few detectable lipid droplets were seen after 3 weeks (Dong et al., 2013; Xu et al., 2013; Hilkens et al., 2014; Lin et al., 2018). Concluding that not only SCAP, but also DPSCs are inferior to BM MSCs when it comes to adipogenic potentiality. It was suggested that they display a rather delayed adipogenicity where even though accumulation of lipid droplets occurs, they do not expand to the expected size of adipocytes (Huang et al., 2009).

**Figure 6:**
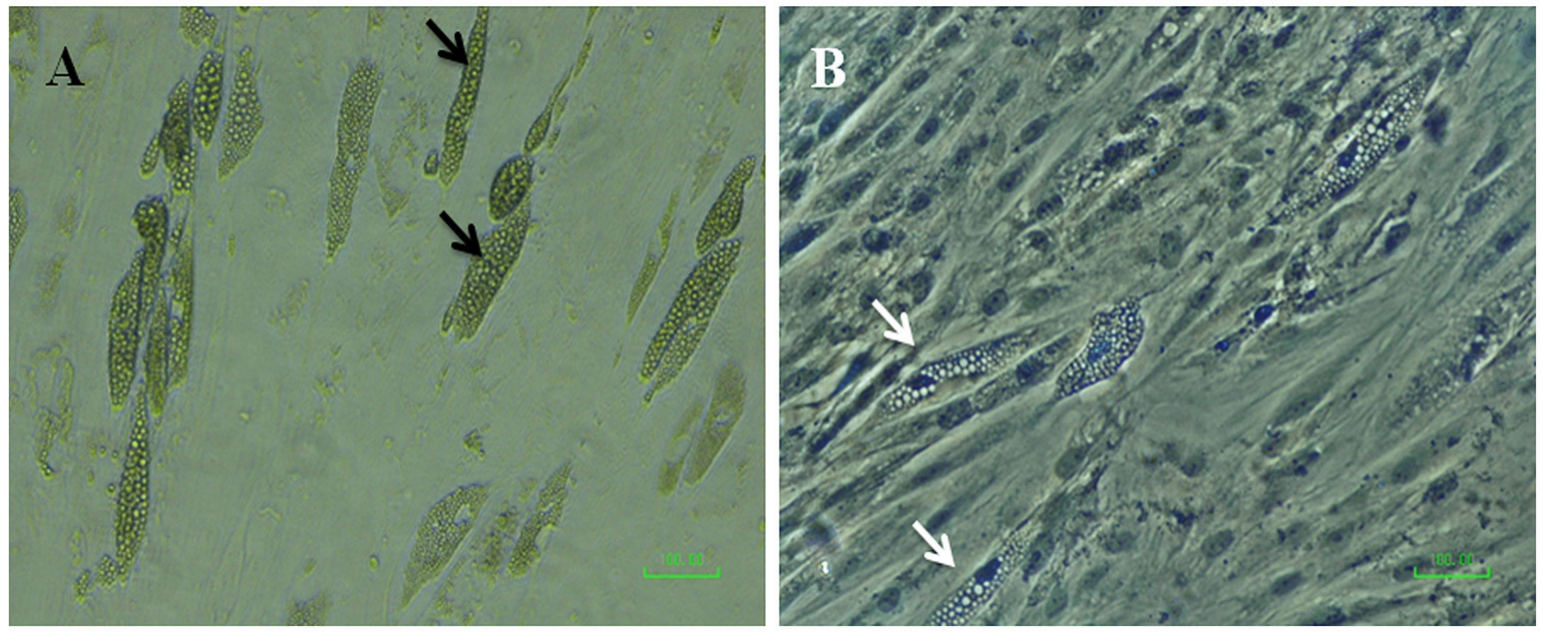
Micrographs of SCAP induced with adipogenic media after 21 days; **(A)** before and **(B)** after Oil Red O staining (with hematoxylin as a counterstain). Black arrows point to possible clusters of oil droplets, however, the white arrows denote the absence of oil red stain uptake by the hypothetical oil droplets.

Despite the fact that previous literature does display SCAP as being capable of adipogenic differentiation, we have found that SCAP obtained from either premolar or wisdom teeth had very weak or a complete lack of adipogenic differentiation potential in spite of the use of a multitude of adipogenic induction protocols (Figure 6). Perhaps, this owes to the limited evidence present amongst previous studies, where even though lipid droplets have been seen to form, their assessment was quite variable among studies in contrast to the more constant results obtained from the osteogenic differentiation experiments (Ding et al., 2010; Xu et al., 2013).

Therefore, we could not ascertain the adipogenic potential of SCAP to be in accordance with previous studies. Nevertheless, there are many factors and limitations to be considered. For instance, a major limitation was the lack of employment of lipophilic gene expression to confirm the lack of adipogenesis. Especially since studies that presented with successful adipogenic differentiation proved so by the positive expression of genes such as LPL (Ding et al., 2010). Moreover, the negative adipogenesis may have been due to the individual sample variation, passage number, protocols used, the stain preparation, or the nature of the cells themselves.

Sonoyama did show typical oil droplets stained red, however the frequency of their occurrence was not mentioned whether or not it was a discrete rather than a uniform oil droplets production (Sonoyama et al., 2008; Xu et al., 2013)While some studies reproduced the same qualitative results, others failed to or showed different qualitative results that were also regarded as lipid droplets, in spite of not being stained red when the oil red O stain was employed, similar to the results that we found. Therefore, criteria that are more definite need to be set in order to define successful adipogenesis.

Moreover, the form of the oil droplets obtained from some wisdom cultures were in the form of clusters which was not however previously reported in that fashion, rather larger, sole, and discrete droplets were described before. This fact coupled with the lack of oil red O stain uptake led us to believe that SCAP do not have a distinct adipogenic potential. Furthermore, the premolar cultures yielded very sparsely and discretely red stained areas that do not have an oily appearance at all, denoting a great uncertainty in the adipogenic lineage. Nevertheless, compared to BM MSC and DPSCs, SCAP have been in fact described as being inferior in adipogenic differentiation potential (Huang et al., 2009; Bakopoulou et al., 2011; Dong et al., 2013; Xu et al., 2013). Hence, it can be at least concluded that in case of targeting, adipogenesis, SCAP should not be the first option considered.

### Chondrogenic differentiation potential

SCAP showed a potential to differentiate along the chondrogenic lineage with successful Alcian Blue staining of proteoglycans produced after a 3 week exposure period to inductive media (Cao et al., 2013). Nevertheless, to our knowledge, no report of chondrogenic gene expression has been documented.

## Extra-classical tri-lineage mesenchymal potential; angiogenesis & neurogenesis

Vascularity and innervation are two properties that cannot be sacrificed when considering tissue regeneration. Particularly when regenerating parts of the tooth due to the limitation of the apical foramen, leads to a dire need for ingrowth of nerve fibers and blood vessels from the apical tissues (Huang et al., 2009).

### Neurogenic potential

Stem cells in general have proven to play a role in the demand supply chain, where needed factors have been found to be supplied when demand takes place due to situations such as tissue injury/repair. This can be useful for the smaller scale of regenerative endodontics, where demand leads to the elaboration of critical factors important for pulpal innervation such as brain-derived neurotrophic factor (BDNF), up to the larger more vital scale of treating neurodegenerative diseases such as Alzheimer's and Parkinson's diseases (de Almeida et al., 2014).

Speaking of neurogenesis and based on the neural crest origin of SCAP, it is only natural to attempt and employ them for neural tissue regeneration (de Almeida et al., 2014). Research has shown that SCAP may indeed possess a neuroprotective role by reducing neuro-inflammation and triggering oligodendrocyte differentiation (De Berdt et al., 2018). Besides the aforementioned MSC markers, SCAP also express neuronal markers. Not only that, but they also secrete neurotrophic factors and stimulate neurite outgrowth. Moreover, when SCAP is subjected to hypoxic conditions, expression of various growth factors and neurospecific genes are upregulated (De Berdt et al., 2015).

SCAP are particularly relevant and significant in regenerative endodontic procedures since they are the cells suggested to populate the root canal area following regenerative endodontics. Furthermore, they have been proven to partake in the processes of neurite outgrowth and axonal targeting, both *in vitro* and *in vivo* (de Almeida et al., 2014).

Despite initially measuring up DSCs against BMMSCs, DSCs seem to have taken the lead in neurogenic differentiation due to their neural crest history which labels them as being originally acquired from neural tissues (Huang et al., 2008). Therefore, the fact that SCAP have a neural crest origin makes them more likely to commit along the neurogenic differentiation line when stimulated appropriately. Therefore, upon neurogenic induction, morphological changes in SCAP become evident, where they start to resemble neurocytes, with spindle shaped bodies and long cellular processes rather than spherical cell bodies with multiple cellular extensions as portrayed by the BMMSCs (Bakopoulou et al., 2013, 2015; Yuan et al., 2014).

Like any other developmental process, dental innervation is orchestrated by a sequence of molecular signals, signals of which are controlled by a collection of dental pulp specific neurotropins such as nerve growth factor (NGF) and BDNF. Dental pulp is innervated by rich trigeminal nociceptive primary afferents. Making innervation of dental tissues particularly interesting is the fact that it becomes disrupted and therefore is rather dynamic as deciduous teeth are shed and permanent teeth erupt. This dynamic fact further supports and renders the idea of regaining innervation as part of regenerative endodontic procedures by making them seem more doable (de Almeida et al., 2014).

Furthermore, a special secretome has been suggested to be released from SCAP following regenerative endodontics procedures, where in response to cold stimuli, they have been suggested to secrete soluble factors that lead to neuronal activation. Fairly interesting, is the fact that this neuronal relationship cannot be due to the hydrodynamic theory seeing as though restorative materials placed post regenerative endodontic procedures involves a cut off of the innervation source coming from the coronal dentinal tubules (Eskander et al., 2017).

In addition, a potentiator of SCAP's neurogenic ability arises by co-culturing them with trigeminal neurons where they react by releasing BDNF, NGF, and glial cell-derived neurotrophic factor (GDNF), therefore labeling SCAP as a chemical mediator of not only neurite outgrowth, but also neuronal survival and overall proving that the immediate microenvironment greatly influences cell behavior. Nevertheless, BDNF alone has been proven to be solely responsible for neurite overgrowth (de Almeida et al., 2014).

Moreover, STRO-1 positive subpopulation of SCAP, as well as neurogenically induced SCAP were found to positively express neurogenic markers, such as beta III tubulin, GAD, neuronal nuclear protein (NeuN), nestin, glial fibrillary acidic protein (GFAP), neural cell adhesion molecule (NCAM), and neurofilament (Sonoyama et al., 2006; Bakopoulou et al., 2013, 2015; Yuan et al., 2014). On an interesting note, Nestin positive MSCs obtained from human embryonic stem cells (ESCs), have been found to differentiate into the 3 germ layers, despite negatively expressing STRO-1 (Lv et al., 2014). This calls for further exploration of the impact of STRO-1 positivity on the neurogenic potential of SCAP, seeing as though they have been classified as mesenchymal stem cells. Nevertheless, again their unique ectomesenchymal nature is probably where the difference lies. Perhaps this calls for a future redirection in classifying SCAP as simply being MSCs, seeing as they are proving themselves to be even more superior.

### Angiogenic potential of SCAP and their role in pulpal regeneration

A common and rather interesting finding among the different stem cell niches is the fact that regardless of the source, whether it is dental or extra dental, stem cells are almost always found to reside in richly vascularized areas. Therefore, and since stem cells have the innate ability to regenerate their original microenvironments, this ensures a solid and predictable role in angiogenic regeneration (Chrepa et al., 2017). SCAP have been found to express a panel of angiogenic proteins; such as vascular endothelial growth factor (VEGF), basic fibroblast growth factor (bFGF), angiopoietin-1, matrix metalloproteinases, endostatin, thrombospondin-1, insulin-like growth factor binding protein 3 (Hilkens et al., 2017).

The pulpal blood supply is threatened long before complete pulp damage, meaning that even when trauma or inflammation affects the tooth, the neurovascular bundle becomes disrupted leading to ischemia of the pulpal tissues (Yuan et al., 2014).

Therefore, revascularization is needed for physiological tissue formation and regeneration (de Almeida et al., 2014). Since SCAP are regarded as MSCs, and MSCs have been found to occupy the body as pericytes or perivascular cells, they can serve as candidates for revascularization. Moreover, the perivascular zone itself is regarded as a niche of MSCs (de Souza et al., 2016). Nevertheless, pericytes are rather difficult in their identification. Clues to their identification owes to their anatomical location of occupying the abluminal surface of endothelial cells in microvasculature. Interestingly, it has been suggested that when an injury occurs, MSCs functioning as pericytes may be delivered from their niche and release immunoregulatory and trophic bioactive factors. Moreover, the reason why pericytes have become more and more recognized as MSCs, is due to the common finding of blood vessels in the majority of tissues where MSCs have been identified. This can relate back to early development, when the mesenchymal tissue functioned to fill spaces prior to the development of the vascular system (Lv et al., 2014).

The dental pulp is primarily made up of a richly innervated and vascularized soft connective tissue surrounded by unyielding hard dental tissues. Routine therapy to a diseased pulp is root canal treatment where the pulp is completely extirpated and replaced by an inert artificial material. Some cases of diseased pulps however involve teeth with immature roots and therefore treatment in these cases should enable root development to proceed and not retard the process. Nevertheless, intervention that promotes apexogenesis might interfere with normal pulp physiology and lead to pulp necrosis followed by cessation of root development. Drawbacks such as root weakening and fracture are natural to follow (Hilkens et al., 2014).

A common limitation of regenerating the pulpal organ is its single source of blood supply through the apical foramen. In healthy pulps, the tight apical foramen supplies 80% of the whole blood supply of the pulpal tissue (Hilkens et al., 2014). Besides the fact that the pulp has the apical foramen as a single main source of blood supply, the actual size of the apex itself is found to be an important factor that governs the success of revascularization. It has been implicated that an optimal apical size of 1.1–1.5 mm increases healing rate and revascularization. Nevertheless, previous studies also showed that implanting DSCs in a tooth with a small apical foramen up to 0.7 mm still led to a successful regeneration of not only organized dental tissues, but also tissues that are vascularized. Additionally, revascularization procedures have been found to be successful when the apical foramen was as narrow as 0.5 mm, nevertheless, the influence of the apical diameter and age cannot be undermined (Hilkens et al., 2014; Estefan et al., 2016). Indeed, the presence of SCAP has been deemed the major reason for successful regeneration of the dentinopulpal organ in previously necrotic immature teeth. As previously mentioned, SCAP can maintain their proliferation and differentiation capacities even in case of pre-existing periapical pathosis. This is in addition to their highly angiogenic secretome which may further promote the revascularization process. Along with the induced bleeding from the apical tissues, SCAP travel along with the blood to settle in the blood clot created inside the disinfected canal thereby contributing to the regeneration process of the dentinopulpal organ and promoting the maturation of the tooth (Lovelace et al., 2011).

#### Clinical goal; exploiting the *in Vivo* behavior of SCAP to trigger endogenous dental regeneration in regenerative endodontics (homing and recruitment strategies)

Recently, the notion of employing growth factors to favor root completion is on the rise. The focus of endodontic regeneration should be to employ the available resources in endodontic regeneration or repair. Regenerative endodontic therapy has been defined as biologically based procedures designed to replace damaged structures, including dentin and root structures, as well as cells of the pulp-dentin complex (Murray et al., 2007)^.^ Therefore, since SCAP are one of the main resources, their functionality needs to be understood under different situations. The notion of regenerative endodontics primarily involves the main resource of stem cells which is brought about by inducing bleeding from the site where the desired stem cells are present, which is peri-apically (Chrepa et al., 2017). This can be achieved post root canal disinfection by inducing a blood clot that is known to bring along with it a release of growth factors and thereby attract the residing stromal cells to the apex. Other suggested alternatives involve the use of scaffolds and angiogenesis promoting growth factors such as VEGF and bFGF; strategies that rely on cell homing (Hilkens et al., 2014; He et al., 2017; De Berdt et al., 2018)^.^ Cell homing-based strategies targeting SCAP have also identified chemotactic factors such as stromal cell-derived factor 1 (SDF-1), transforming growth factor beta 1 (TGF-b1), granulocyte-colony stimulating factor (G-CSF) and particularly the combination of the latter two factors as future targets for clinical application as they enhanced both the migration and differentiation potential of SCAP *in vitro* (Fayazi et al., 2017).

Moreover, the choice of scaffolds is also an important factor that needs to be taken into consideration when attempting dentin-pulp regeneration. Despite the well-established similarities between bone and dentin, one can be inclined to choose a scaffold with osteoinductive properties to ensure dentinogenesis. However, this would contradict the needs of dentin pulp regeneration, since both dentin and pulp are governed by certain locations. Therefore, if a scaffold with osteoinductive properties such as tricalcium phosphate (TCP) and hydroxyapatite (HA) were to be used, a risk of generalized calcification could occur throughout the pulp space (Huang et al., 2009). Therefore, greater care must be taken when aiming to regenerate the dentino pulpal organ.

#### Paracrine and secretory profile of SCAP (Table 2)

Advanced pulp necrosis and particularly cases with apical periodontitis pose as a threat to the health of the apical papillae involved. Despite such chronic pathologies SCAP have demonstrated an ability to not only remain vital but also thrive in such situations. Interestingly and in accordance to other studies (Shi and Gronthos, 2003), SCAP obtained from inflamed tissues exhibiting increased vascularity portray enhanced angiogenic potential (Chrepa et al., 2017).

**Table 2:** SCAP biological relevance in terms of differentiation and/or paracrine potential with references included.

**SCAP differentiation potential**	***In vivo/in vitro***	**Cell secretome/ implanted cells**	**Factors involved**		**Ref**
Osteogenic/ odontogenic	Both	Implanted cells	DSPP BSP ALP BGLAP BMP RUNX2		Bakopoulou et al., 2013
Adipogenic	*In vitro*	Implanted cells	LPL PPAR gamma2		Ding et al., 2010; Bakopoulou et al., 2013
Chondrogenic	*In vitro*	Implanted cells	Proteo-glycans		Cao et al., 2013
Neurogenic	Both	Cell secretome GDNF NT3 CNP Survivin NSE VEGFA BFGF	BDNF NGF GDNF GAD NeuN Nestin GFAP NCAM Neuro-filament		Sonoyama et al., 2006; Bakopoulou et al., 2013, 2015; de Almeida et al., 2014; Yuan et al., 2014; Germain et al., 2015
Angiogenic	Both	Cell secretome bFGF PDGF VEGF angiopoietin-1 MMPs Endostatin IGFBP-3 ephrinB2 HIF-1	+ uPA, EDN2 DPPIV ANG	– PAI-1 THBS1 TIMP1/4 PTX3 PGGF PAO-1	Hilkens et al., 2017 Yuan et al., 2014

MSCs are particularly unique in reference to their metabolic flexibility where they have the ability to withstand anaerobic conditions, through the use of glycolic rather than mitochondrial respiration. Moreover, tissue damage or systemic stimuli such as hypoxia or inflammation act as angiogenic stimulators through the elaboration of angiogenic stimulants/factors (Bakopoulou et al., 2015). Therefore, it has been suggested that short term hypoxia does lead to not only the stimulation of endogenous angiogenesis, but also aids in avoiding the rejection of transplanted tissues, as well as acts to exclude the overall possible degeneration of the tissues involved, thereby maintaining the tissue at hand (Yuan et al., 2014).

Moreover, when compared to follicle precursor cells (FSCs), both DPSCs and SCAP were found to take the upper hand in stimulating the vascularization of regenerated dental tissues (Hilkens et al., 2014). This could owe to their close proximity to insults or injury since generally, DSCs express paracrine angiogenic factors such as VEGF, bFGF, and platelet derived growth factor (PDGF) when subjected to a state of injury/hypoxia or even under basal conditions. Moreover, SCAP particularly also secrete ephrinB2, and HIF-1 (Hilkens et al., 2014; Yuan et al., 2014). The expression patterns of both HIF1 alpha and EphrinB2 confirm that hypoxia stimulates the liberation of factors that lead to angiogenesis, simply implying that where there is a demand, comes a supply.

Furthermore, the apical papilla can be taken advantage of in achieving angiogenesis due to its location in the perivascular niche, as well as its expression of CD146 and STRO-1 and also since it has been found to possess a positive expression of VEGF throughout the entire tissue (Hilkens et al., 2014; Bakopoulou et al., 2015). Out of the specific growth factors important and relevant to angiogenesis, SCAP secretes Angiopoietin-1 (ANGPT-1), IL-GF binding protein 3, and VEGF at the following respective levels; 7503, 666.3, and 1482 pg/ml (Hilkens et al., 2014).

Moreover, the paracrine angiogenic potential of SCAP at an mRNA level, can be seen to secrete both classes of angiogenic stimulating and inhibiting factors, such as uPA, EDN2, DPPIV, ANG, and PAI-1, THBS1, TIMP1/4, PTX3, and PGGF respectively. Additionally, SCAP hold potential for endothelial transmigration through the elaboration of factors such as ANGPT1, EDN1, IGFBP43, uPA, and VEGF. In conclusion, through further assays such as chorioallantoic membrane assay, SCAP have proven to be superior to DPSCs and human gingival fibroblasts (hGF) in terms of their overall angiogenic potential (Hilkens et al., 2014). Under hypoxic conditions, SCAP were tested for its angiogenic potential in the presence of human umbilical vein endothelial cells (HUVECs) at a ratio of 1:5. The act of co-culturing with SCAP, that already possess a robust angiogenic potential, potentiated previously committed endothelial cells and led to establishment of successful tubulogenesis (Yuan et al., 2014).

Moreover, normally, when cells are not supplied by the needed factors to thrive, they tend to liberate them themselves, yielding a secretion known as the cells' secretome; which also entails secreted trophic and immunomodulatory cytokines (Bakopoulou et al., 2015). This secretome has the potential of being of valuable importance according to its content. For instance, the secretome from MSCs has a therapeutic indication in cardiovascular diseases, traumatic brain injuries, chronic wounds, and bone defects (Bakopoulou et al., 2015). The secretome content is however not as predictable as it ideally would be, it is very easily fine-tuned based on the cell's surroundings. Nevertheless, this fine tuning can speak volumes and lead to a significant variation in the secretome's effect. For instance, SCAP subjected to serum deprivation released pro and anti-angiogenic factors that induced the migration of endothelial cells and tubule formation. Furthermore, they induced new blood vessel formation (Bakopoulou et al., 2015).

Moreover, the combinatory elimination of all 3 variables; serum, oxygen, and glucose at once were actually found to mimic an *in vivo* condition of severe ischemia. In spite of that, SCAP morphology and viability remained rather unchanged reflecting their striking adaptability. Moreover, this combinatory elimination actually yielded greater proangiogenic factors and lesser antiangiogenic ones, leading to an overall superior angiogenic paracrine activity and endothelial transdifferentiation potential, classifying it as the most favorable condition vs. serum and oxygen deprivation condition or oxygen deprivation alone. All the aforementioned qualities make SCAP an appealing candidate for diseased and ischemic tissues (Bakopoulou et al., 2015).

To conclude, SCAP, as well as DPSCs seem to be the finest candidates for angiogenesis, since they demonstrated noteworthy improvement of the process of neoangiogenesis. Denoting that even with increased elaboration of angiogenesis inhibiting factors; such as tissue inhibitors of metalloproteinases (TIMP), plasminogen activator inhibitor-1 (PAO-1), thrombospondin-1 (THBS1), and long pentraxin (PTX3), pro angiogenic factors seem to have taken the lead (Hilkens et al., 2014).

## Paracrine and secretory activity; implications for inflammation and infection

Generally, adult MSCs *in vivo* are found to be non-proliferative and in a quiescent phase until properly stimulated by signals triggered by tissue damage and re-modeling (Sonoyama et al., 2008). By mimicking their original microenvironment or niche, SCAP obtained from inflamed tissues do show not only an increase in proliferation but also in their osteogenic potential (Liu et al., 2016; Chrepa et al., 2017). For instance, when exposed to inflammatory mediators that are likely to be expressed in cases of infected immature teeth such as tumor necrosis factor-alpha (TNF-α), interleukin-1 beta (IL-1β), properties of SCAP surely differ. Moreover, SCAPs properties differ in a way where these factors and mediators come to have an inhibitory effect on SCAPs osteogenic/dentinogenic differentiation ability. Nevertheless, other confounding variables have been suggested, such as the duration of the exposure/ contact time of SCAP with these inflammatory cytokines due to the intricate signaling pathways involved. Such variables include contact time, where short contact time acts as a potentiator, while longer contact time acted as a retarder of differentiation properties. The short term potentiating effect could owe to the initial defensive and robust reactionary state of SCAP to a sudden stimuli/state of injury that later becomes attenuated as the contact time increases (Liu et al., 2016).

Further proof in favor of SCAP's perseverance is the fact that after endodontic disinfection in cases with periapical pathologies, the survival of both SCAP and ERSH was evident by the successful differentiation of 1ry odontoblasts (Sonoyama et al., 2008).

Therefore, even though the health of the apical papilla can be easily threatened in case of advanced pulp necrosis and particularly in situations with apical periodontitis, SCAP have demonstrated an ability to not only remain vital but also thrive in such situations. Interestingly and in accordance to other studies, SCAP obtained from inflamed tissues that usually exhibit increased vascularity portray enhanced angiogenic potential (Shi and Gronthos, 2003; Chrepa et al., 2017). Once more, mimicking their original microenvironment in a favorable manner. This could classify SCAP as being able to make a perfectly favorable result from an imperfect situation.

## Applications of SCAP *in Vivo*

When considering functional regeneration, a major success contributor lies in the root. Seeing as though it is the root that supports the crown, be it artificial or natural. Therefore, without a solid base of a root, the crown would fail to achieve the desired tooth function. Sonoyama et al. were able to utilize human SCAP to develop a functional biological root (bio root) in a swine model (Sonoyama et al., 2006; Huang et al., 2008; Wang et al., 2013).

Moreover, dental pulp diseases are known to be inevitable in the majority of patients presenting in dental clinics. Conventional therapy continues to be less than perfect. The ideal therapy to preserve teeth in a healthy state would be to regenerate the dentin-pulpal organ with sufficient vascularity.

Compared to stem cells from human exfoliated deciduous teeth (SHED) and DPSCs, SCAP have the upper hand in *de novo* dentin regenerative potential, most probably owing once more to their origin being from a developing tissue. Nevertheless, with accordance to SHED and DPSCs the apparent mechanism for the *de novo* dentin pulpal regeneration by SCAP can be broken down to the following steps:

1. SCAP migrating onto the dentin surface.
2. Dentin releases embedded growth factors, that act as signals for cellular differentiation into odontoblast like cells.
3. The cellular processes of each differentiated cell extend into the dentinal tubules.
4. Differentiated cells produce ECM onto the dentin surface and into the dentinal tubular spaces.

The steps that follow in the formation of dentin like tissues, are then similar to the natural dentinogenesis. Nevertheless, a difference lies in the pace of formation of the regenerated dentin. To have a solid foundation regarding *de novo* regeneration, higher order animals need to be experimented on (Huang et al., 2009).

Moreover, SCAP are subject to many implications in terms of their superior neurogenic functionality as cells existing in a niche or microenvironment of neural crest origin. These implications were fulfilled in a study that proved improved function and tissue regeneration when the apical papilla as a whole was transplanted in a spinal cord injury in rats. The use of SCAPs inherent scaffold i.e., its original niche offered maintenance of post transplantation integrity that was not offered by synthetic scaffolds, where a non-favorable lesion environment takes the upper hand (De Berdt et al., 2015). Furthermore on the relation of SCAP to its neural crest origin, is the neural marker Nestin which was found to be expressed at a level higher than 99% in the original heterogeneous population (Bakopoulou et al., 2013) indicating that SCAP are in fact more or less unaltered from their neural crest origin.

Besides SCAP's inherent niche, fibrin hydrogels (Fibrinogen 50-Thrombin50) were found to promote neurogenic differentiation. This suggests similarities between fibrin and the extracellular matrix; where it not only has mechanical advantages, but also promotes differentiation and elaboration of angiogenic factors (Germain et al., 2015). Moreover, enhancement of SCAPs viability is noted when supplementation of hydrogels with a hyaluronic acid based foundation is performed; where the combination yields a better quality scaffold; in terms of surface and mechanical features. Hyaluronic acid based hydrogels were found to enhance the proliferation rate, metabolic activity, and collagen producing ability of SCAP, while depressing their apoptotic activity (Lambricht et al., 2014).

Moreover, properties of scaffolds can be adjusted by preincubating them in media prior to cell seeding. A study using 3D silk fibroin as a natural scaffold tested the effect of preincubation on cellular attachment and proliferation, where post seeding, enhancement of the attachment and proliferation of SCAP was noted. Preincubation resulted in scaffold modifications in the form of reductions in surface roughness and the water contact angle. Moreover, the duration of the preincubation period was proportional to the enhancement of the properties (Amirikia et al., 2017). Other natural scaffolds that enhance cellular activities of SCAP are chitosan–based scaffolds. Further enhancement of cellular activities was noted when the chitosan-based scaffold was used as a vehicle for release of growth factors such as TGF-b1. Relevantly, the release of TGF-b1 led to a natural improvement in SCAPs odontogenic differentiation potential (Bellamy et al., 2016). In the same manner, tailoring of regenerative therapy can be targeted according to the desired line of differentiation.

The existing literature has therefore highlighted the lack of functional efficiency of isolated SCAP, which might have been either possibly brought about by cellular modifications that occur due to plastic culture interference or due to the inadequacy of the synthetic scaffold used *in vivo*. Hence, relating the importance of the mode of delivery to the condition of the cells, and concluding that the original niche of any cell would function as the most favorable microenvironment/scaffold (De Berdt et al., 2015). Further work is needed to learn more about the secretory activity of the transplanted SCAP.

## SCAP preservation

### *In Vivo* SCAP preservation

More often than not, research is too far from the patient's immediate benefit. However, this is not always the case and therefore, clinicians need to be constantly updated about both the dental materials and techniques they use and how they can directly affect various tissues in the tooth, even if those tissues are out of sight, such as the case with the apical papilla.

Commonly used dental filling materials are composed of resinous monomers. Even though these fillings might be confined to the crown and despite the fact that they can be underlined by an intact dentin barrier, their potential cytotoxicity is well documented. This is due to the permeable nature of the dentinal tubules, where their permeability acts as a gateway to the pulpal tissues. The soft connective tissue nature of the pulp would further permeate molecular diffusion through the apex and in case of an open apex, onto the apical papilla tissue. Consequently, enabling the transmission of residual substances to distant dental tissues. Such residual substances arise from the utilized resin based materials due to either their incomplete polymerization at the time of placement or due to time related degradation of the resin material itself (Bakopoulou et al., 2012). It is therefore important that clinicians be aware of the effects of the materials being used especially in cases presenting with immature roots. Furthermore, understanding the effects of dental materials is important in order to reach a dental material that preferentially stimulates dental tissue regeneration.

Components that make up the very commonly used dental composite resin fillings; such as Tetraethyleneglycol Dimethacrylate (TEGDMA) and Hydroxyethyl methacrylate (HEMA) have been proven to inversely affect the properties of SCAP, such as their viability, odontogenic differentiation potential and mineralization potential. Furthermore, it was concluded that HEMA is more toxic to SCAP at a lower concentration (0.05, 0.1 mM), while TEGDMA is considered to be more SCAP friendly in terms of differentiation (Bakopoulou et al., 2012).

Furthermore on dental materials affecting the SCAP properties is the mineral trioxide aggregate (MTA). Since it is regarded an osteoinductive dental material, MTA is used in endodontic therapy for various applications such as repair of perforations, apexification, root end fillings or simply as a pulp capping material. As such, MTA is often used clinically in locations involving the apex, rendering SCAP to be the most affected and targeted population of cells, especially when MTA is used after apical bleeding is induced, since SCAP has been shown to occupy perivascular locations. MTA has been therefore specifically known for its regulatory effects on cellular activities (Schneider et al., 2014).

Despite the efficiency of MTA as an osteoinductive material, it can often be unsightly due to its dark shade, labeling it as gray MTA due to its dark hue. For esthetical considerations, white MTA (WMTA) was developed, which differs in its tetracalcium aluminoferrite content. When tested on SCAP, WMTA was actually found to further enhance SCAP properties, however only on an early and short term basis for cellular migration, which was improved from 1st to the 6th hour, while proliferation capabilities were enhanced on a more sustained level, lasting for a duration of 5 days (Schneider et al., 2014). This selectivity of sustained vs. non-sustained properties opens up room for tailoring the targeted treatment at hand.

Moreover, on WMTA, an interesting finding is the effect of fresh WMTA in contrast to aged WMTA, where the latter holds a greater potential for proliferation induction. Whereas, the former led to a release of calcium ions at a toxic level that caused an initial inflammatory reaction leading to an early (day 1) decrease, rather than the expected increase in the proliferation (Schneider et al., 2014). Hence, possibly encouraging the use of aged WMTA.

Besides the effect of dental materials on SCAP, clinical procedures that do not employ dental materials also have an effect on SCAPs properties. Namely, forces (cyclic stress) applied in orthodontic treatment that is composed of 200 g of compressive forces has been found to stimulate the osteogenic/odontogenic differentiation potential of SCAP despite causing the cells to lose their typical stem cell features. This osteogenic preference is possibly related to the cessation of cell proliferation which potentially marks the beginning of specific tissue development. This poses a significance in terms of how the apical structures remodel themselves and are altered to coincide with orthodontic forces. Therefore, since SCAP has a prime role in root maturation and apexogenesis and seeing as though the majority of orthodontic therapies are performed on younger age individuals having immature roots, such findings are essential to be put into consideration to maintain and preserve SCAP in their optimal and targeted clinical condition. (Mu et al., 2014).

The fact that a majority of patients who have been subjected or who will be subjected to regenerative endodontic procedures require orthodontic intervention is an important consideration from the clinical standpoint (Chaniotis, 2018). Indeed, although regenerative procedures preceding orthodontic treatment maybe successful and not adversely affect the final treatment outcome; better understanding of the biological influence of these procedures will be required for appropriate future treatment planning.

### *In Vitro* SCAP preservation

One of the fairly common dental wastes is the wisdom tooth. Studies show that 70% of patients at least have one impacted tooth that threatens the health of neighboring teeth and adjacent soft tissues and therefore becomes indicated for prophylactic extraction (Ding et al., 2010). Other common dental wastes are teeth extracted for orthodontic purposes such as supernumerary teeth, or most commonly premolars. At the time of extraction, these dental patients are normally in a younger age range. Therefore, their health state is more likely to be stable and are thereby not in actual dire need of SC therapy. Unfortunately, though, this state of health with the progress of years more often than not turns to disease. Whether this disease is mild or severe, it can surely benefit from the therapeutic effect of stem cells, especially if they are autologous, i.e., from the same patient to himself.

This notion involves using the cells from today for the disease of tomorrow. This can be applied to SCAP, during routine dental extractions. The question lies in whether or not SCAP can withstand the process of banking, which involves a procedure known as cryopreservation, where cells are preserved by being stored in certain solutions under very low temperatures.

Previously conducted studies tackled this issue and did prove that SCAP survive cryopreservation in a remarkable manner. Generally speaking, both fresh and cryopreserved SCAP met Dominici's minimal criteria (2006) and Perry (2008) (Dominici et al., 2006; Perry et al., 2008). In addition, when comparing SCAP before and after cryopreservation, basic stem cell properties such as fibroblastic morphology, cell viability, proliferation and colony forming units; no differences incurred 6 months after cryopreservation. Furthermore, regarding the biological properties of SCAP post cryopreservation, is the expression of odontogenic markers such as DSPP, as well as adipogenic markers such as LPL and peroxisome proliferator activated receptor gamma2 (PPAR gamma2), all of which remained to be positively expressed. Moreover, confirmation of osteogenic and adipogenic potentiality via von kossa and oil red O staining also remained positive after cryopreservation. Regarding SC markers, both CD146 and STRO-1 were positively expressed as well (Ding et al., 2010).

These findings give a rather promising indication that SCAP are more or less resilient. Further studies need to test this theory, however over longer periods of time that would mimic a life time for a patient who preserved his/her cells at a young age to be used in an older age. This however in itself may not be entirely necessary since the immunogenic properties of SCAP found to acquire low immunogenicity and immunosuppressive functions (Chen et al., 2012). Thereby, opening up options for allogeneic use of cryopreserved SCAP and therefore nullifying the need to test cryopreservation of SCAP for life span periods.

## Future research directions

Recently, light has been shed over extracellular vesicles of dental origin, suggesting more potent osteogenesis when exposed to BM MSCs, however, SCAP has not been studied for this aspect yet. (Alraies et al., 2017) Therefore, a deeper exploration of the SCAP associated extracellular vesicles would definitely add to potential translational therapy.

In reference to the angiogenic properties of SCAP, research can be conducted to find a correlation between the expression of VEGF and ephrin B2, and more specifically how to increase the protein expression of ephrin B2 and not just the mRNA level (Yuan et al., 2014).

On a greater scales, the *in vivo* angiogenic potential of SCAP and their dynamic secretome need to be further explored (Bakopoulou et al., 2015).

Perhaps the question that actually remains is whether or not root apical papilla can be successfully cryopreserved and thawed as a whole tissue for cell-based therapy. Since this would make handling of SCAP much more convenient, time effective, and less demanding therefore, encouraging the idea of banking altogether. Studies have shown that teeth can be banked in total for successful retrieval and culture of DPSCs without adversely affecting their innate *in vitro* potentials (Perry et al., 2008).

Moreover, despite the alleged direct relation between the absence of the apical papilla and failure of apexogenesis, further research is necessary to determine that is the absence of the apical papilla itself and not the epithelial root sheath of Hertwig that causes a failure in root development (Huang et al., 2008). Therefore, the clear role of SCAP in root development needs to be elucidated, is it the ERSH, the apical papilla, or periodontal ligament stem cells (PDLSCs) that become isolated in culture?

Nevertheless, these SCAP continue to prove themselves capable of being an excellent source of not only mesenchymal stem cells but also ectodermal stem cells, owing to their neural crest origin. Therefore, a huge amount of dedication to SCAP research is due in order to utilize these readily available, conveniently obtained, often waste bound stem cells that lie in each and every oral cavity at a certain moment in time. Hence, they should be targeted for maximum benefit of stem cell research and translational medicine.

## Ethics statement

All experiments were performed in accordance with the Ethics Research Committee at both the Faculty of Dentistry, Alexandria University and the university medical center Hamburg, respectively.

## Author contributions

ON performed the experiments, analyzed and interpreted the data. As well as drafted initial version of the manuscript. RE devised the project outline, analyzed and interpreted the data. As well as drafting the initial outline of the manuscript in addition to revising it critically for important intellectual content. Both authors approved the final version to be published and are accountable for all aspects of the work.

### Conflict of interest statement

The authors declare that the research was conducted in the absence of any commercial or financial relationships that could be construed as a potential conflict of interest. The reviewer SB and handling Editor declared their shared affiliation.

## Abbreviations

ALP: Alkaline phosphatase
AM: Amniotic membrane
ANGPT-1: Angiopoietin-1
CFE: Colony forming efficiency
BDNF: Brain-derived neurotrophic factor
bFGF: Basic fibroblast growth factor
BGLAP: Bone gamma-carboxyglutamate protein
BM MSC: Bone Marrow Mesenchymal Stem Cell
BMP: Bone morphogenetic protein
BSP: Bone Sialoprotein
DPSCs: Dental pulp stem cells
DSC: Dental stem cell
DSP: Dentin Sialoprotein
DSPP: Dentin sialophosphoprotein
ERSH: Epithelial root sheath of Hertwig
ESCs: Embryonic stem cells
FBS: Fetal bovine serum
FSCs: Follicle precursor cells
GAD: Glutamic acid decarboxylase
G-CSF: Granulocyte-colony stimulating factor
GDNF: Glial cell-derived neurotrophic factor
GFAP: Glial fibrillary acidic protein
HA: Hydroxyapatite
HEMA: Hydroxyethyl methacrylate
HGF: Human gingival fibroblasts
HIF-1: Hypoxia-inducible factor-1
HUVECs: Human umbilical vein endothelial cells
IL-1β: Interleukin-1 beta
IGF-1 & 2: Insulin like growth factor 1 & 2
LPL: Lipoprotein lipase
MPNST: Malignant peripheral nerve sheath tumor cells
MSC: Mesenchymal Stem Cell
MTA: Mineral trioxide aggregate
NCAM: Neural cell adhesion molecule
NeuN: Neuronal nuclear protein
NGF: Nerve growth factor
OCN: Osteocalcin
OSX: Osterix
PAO-1: Plasminogen activator inhibitor-1
PDGF: Platelet derived growth factor
PDLSCs: Periodontal ligament stem cells
PL: Platelet lysate
PPAR gamma2: Peroxisome proliferator activated receptor gamma2
PTX3: Long pentraxin
RUNX2: Runt-related transcription factor 2
SCs: Stem Cells
SCAP: Stem cells from the apical papilla
SDF-1: Stromal cell-derived factor 1
SHED: Stem cells from human exfoliated deciduous teeth
TCP: Tricalcium phosphate
TEGDMA: TetraethyleneglycolDimethacrylate
TGF-b1: Transforming growth factor beta 1
THBS1: Thrombospondin-1
TIMP: Tissue inhibitors of metalloproteinases
TNF-α: Tumor necrosis factor-alpha
VEGF: Vascular endothelial growth factor
WMTA: White MTA.
